# Evaluation of the effects of additional therapy with *Berberis vulgaris* oxymel in patients with refractory primary sclerosing cholangitis and primary biliary cholangitis: A quasi-experimental study 

**Published:** 2021

**Authors:** Zahra Naghibi, Hassan Rakhshandeh, Lida Jarahi, Seyed Mousalreza Hosseini, Mahdi Yousefi

**Affiliations:** 1 *Department of Persian Medicine, School of Persian and Complementary Medicine, Mashhad University of Medical Sciences, Mashhad, Iran*; 2 *Pharmacological Research Center of Medicinal Plants, Mashhad University of Medical Sciences, Mashhad, Iran *; 3 *Department of Community Medicine, Faculty of Medicine, Mashhad University of Medical Sciences, Mashhad, Iran*; 4 *Department of Internal Medicine, Faculty of Medicine, * *Mashhad University of Medical Sciences, Mashhad, Iran*; 5 *Surgical Oncology Research Center, Mashhad University of Medical Sciences, Mashhad, Iran*

**Keywords:** Primary sclerosing cholangitis primary biliary cholangitis Berberis vulgaris, Cholestasis, Alkaline phosphatase

## Abstract

**Objective:**

There are several studies reporting the therapeutic effects of *Berberis vulgaris* on liver diseases. This study was done with the purpose of examining the effect of *B. vulgaris* oxymel (BO) in patients with refractory primary sclerosing cholangitis (PSC) and primary biliary cholangitis (PBC), who did not respond to current treatment.

**Materials and Methods:**

Patients with PSC or PBC who were receiving ursodeoxycholic acid (UDCA, 13-15 mg/kg/day) for at least six months, but their serum levels of alkaline phosphatase (ALP) were still 1.5 folds higher than the normal upper limit during the last six months, were asked to participate in this quasi-experimental study. Patients were asked to take 0.5 ml/kg/day of BO two times a day for three months along with UDCA. At the end of the study, serum levels of ALP, aspartate aminotransferase (AST), alanine aminotransferase (ALT), gamma-glutamyl transferase (GGT), total bilirubin (TB), direct bilirubin (DB), and creatinine as well as prothrombin time (PT), international normalized ratio (INR) and quality of life (QOL) based on PBC-40 questionnaire were assessed as outcomes.

**Results:**

Our results showed that BO notably attenuated the serum levels of ALP, AST, ALT, GGT, TB, and DB, as well as PT and INR and significantly improved QOL.

**Conclusion:**

For first time, we showed that additional therapy with BO has a promising effect in the treatment of refractory PSC and PBC.

## Introduction

Liver failure, liver cirrhosis, and liver transplantation or even related concluding deaths are complications of primary biliary cholangitis (PBC) and primary sclerosing cholangitis (PSC). Several studies indicated that PSC and PBC are considered a chronic autoimmune and multifactorial liver disease with unknown pathogenesis (Kowdley et al., 2018 ▶; Milkiewicz et al., 2012 ▶; Wunsch et al., 2014 ▶). Ursodeoxycholic acid (UDCA) is routinely prescribed for PBC and PSC as a current therapy (Lazaridis et al., 2001 ▶), although many studies emphasize lack of appropriate effectiveness of this medication in patients with PBC and PSC (Angulo et al., 2000 ▶; Corpechot, 2012 ▶; Eaton et al., 2019 ▶). 

In both complementary and alternative medicines, it has been reported that *Berberis vulgaris* (*B. vulgaris*) fruit shows several protective effects in the treatment of liver diseases by its anti-inflammatory and antioxidant properties (Abd et al., 2013 ▶; Imanshahidi and Hosseinzadeh, 2008 ▶; Zarei et al., 2015 ▶). In this regard, its therapeutic effects on non-alcoholic fatty liver diseases (Iloon et al., 2015 ▶; Pisonero-Vaquero et al., 2015 ▶; Rodriguez-Ramiro et al., 2016 ▶) and hepatocellular carcinoma (HCC) (Hanachi et al., 2006 ▶) were also described. These studies demonstrated that *B. vulgaris* decreases biochemical markers of the liver damage, and in contrast, improves histopathological changes of the liver in cholestasis and diabetes (Mirazi et al., 2015 ▶; Rahimi-Madiseh et al., 2017b ▶). 

Base on this evidence, we hypothesized that addition of *B. vulgaris *to conventional therapy (UDCA) may be effective in the treatment of patients with refractory PSC and PBC. Therefore, the present study was conducted to investigate the effects of *B. vulgaris* oxymel (BO) on reduction of alkaline phosphatase (ALP) levels in patients with refractory PBC and PSC. 

## Materials and Methods

**Ethical statements**

The study protocol was approved by the ethics committee of Mashhad University of Medical Sciences (IR.MUMS.REC.1396.140). The study followed the ethical standards of the committee responsible for human experimentation (institutional and national), and with the Helsinki Declaration of 1975, as revised in 2008. This trial was registered on the Iranian Registry of Clinical trials (IRCT2017082435883N1). After the enrollment and before starting the study, all procedures and the objectives and possible benefits and side effects were clearly explained by the researchers to the respected patients. Afterward, the informed consent forms were freely completed and signed by all patients. 

**Preparation of** ***B. vulgaris*** **fruit extract**

*B. vulgaris* fruits were collected from Birjand (South Khorasan province, Iran) in October 2017 and identified by a botanist (Mrs. Sozani). The collected samples were referenced with the herbarium number 13243 at the School of Pharmacy, Mashhad University of Medical Sciences. The fruits were completely cleared, washed and dried in the shade at room temperature (22-25°C). Next, the fruits were powdered by an electrical grinder. To prepare the hydro-alcoholic extract, the powdered fruits were macerated in aqueous ethanol (70% v/v) for 72 hr and the mixture was frequently stirred (Ghorbani et al., 2016 ▶). Afterward, the mixture was filtered out by a mesh (106 µm pore size) and the volume was decreased by rotary evaporator under reduced pressure at a maximum temperature equal to 40°C. A concentrated extract with a 45% w/w yield (calculated based on the powdered fruits), was obtained and stored in dark glass containers at 4°C.

**Standardization of** ***B. vulgaris*** **fruit extract**

A sample of 20 µl of the extract (10 mg/ml) or gallic acid as the standard (0, 50, 100, 150, 250, and 500 mg/l) was added to 100 µl of Folin-Ciocalteu reagent and 300 µl sodium carbonate solution (1 mol/l). The volume was adjusted to 2 ml by deionized water and after 2 hr, the absorbance was measured by a spectrometer at 765 nm. The total phenol content of the extract was calculated using the standard curve plotted for gallic acid and expressed as milligram of gallic acid equivalents (Hosseini et al., 2017 ▶). The assay was performed in triplicate.

**Preparation of** ***B. vulgaris*** **oxymel**

**Figure 1:**
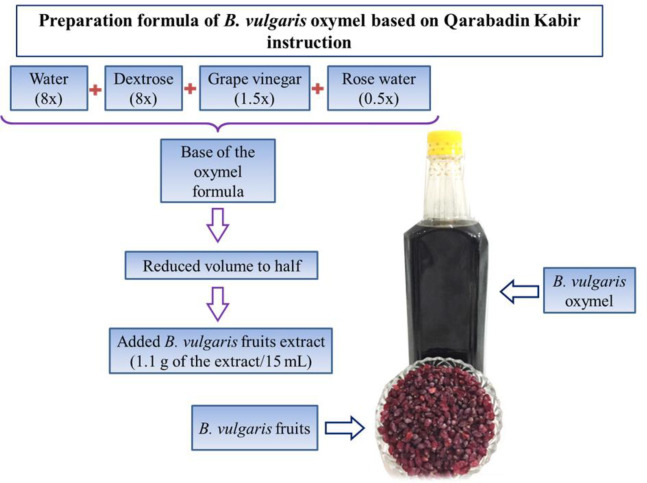
Schematic representation of *B. vulgaris* oxymel preparation

Oxymel is a type of syrup that is prepared using different mixtures of sugar or honey and vinegar. In traditional medicine, it is used alone as a medicine or as a base for the formulation of other medicines (Zargaran et al., 2012 ▶). The preparation formula of the BO was carried out according to the traditional suggested formula taken from the book called “*Qarabadin Kabir*” (khorasani, 1859 ▶). Simple oxymel was prepared by mixing eight volumes of water, eight volumes of dextrose, one and a half volumes of grape vinegar, and half a volume of rose water (8:8:1.5:0.5). The mixture was boiled until its volume was reduced to half. After cooling, the *B. vulgaris *fruit extract was added to the oxymel (1.1 g per 15 ml) to prepare the BO (Figure 1). The product was tested negative for *Salmonella enterica*, *Escherichia coli*, *Pseudomonas aeruginosa*, *Candida* *albicans*, and *Bacillus cereus* contaminations.

**Study design**

This quasi-experimental study was conducted in the gastroenterology department of Imam Reza and Ghaem Hospitals, Mashhad University of Medical Sciences, Mashhad, Iran, from November 2017 to September 2018. Initially, 87 patients with PBC or PSC, were enrolled and then, screened for inclusion criteria; finally, thirty patients (including 15 PSC patients and 15 PBC patients) were recruited for the present study. The sample size was calculated based on the ALP alternation shown in a previous study (Angulo et al., 2008 ▶) and by using the formula for estimating the mean of quantitative variables (ALP) in population.

$N=\frac{{\left(\mathrm{Z\alpha}+\mathrm{Z\beta}\right)}^{2}}{{\left(\frac{\delta }{\sigma }\right)}^{2}}$

Hence, for α=0.05 and β=0.2, 26 persons were considered. We increased the sample size to 30 persons due to a 10% loss of follow up.

**Inclusion and exclusion criteria**

**Inclusion criteria**

The inclusion criteria for enrollment were PBC and PSC patients (based on the diagnostic criteria of the American Association for the Study of Liver Diseases for PSC and PBC) (Chapman et al., 2010 ▶; Lindor et al., 2009 ▶) who were receiving UDCA 13-15 mg/kg/day for at least six months; however, the ALP serum levels were 1.5 folds higher than the normal upper limit (NUL) during the last six months.

**Exclusion criteria**

Exclusion criteria included having other liver diseases, such as congenital or metabolic liver diseases, autoimmune hepatitis, chronic viral hepatitis**, **and model end-stage liver disease (MELD)≥15, pregnancy, cancer, age < 18 years old, diseases that can decrease quality of life (QOL) like severe asthma, kidney dysfunction requiring dialysis, uncontrolled diabetes, severe rheumatoid arthritis, or severe heart dysfunction and increasing liver enzymes, worsening of clinical symptoms as well as occurring one of the mentioned exclusion criteria such as pregnancy during the study.

**Primary and secondary outcomes assessment**

The level of ALP changes was measured as the primary outcome. A demographic data form was completed before the study for each patient. The levels of ALP and other related liver function biomarkers associated with the cholestasis including aspartate aminotransferase (AST), alanine aminotransferase (ALT), gamma-glutamyl transferase (GGT), total bilirubin (TB), direct bilirubin (DB), prothrombin time (PT) and international normalized ratio (INR) were measured by a blood test before starting the study and monthly during the study during a three-month follow-up period using BT3500 auto analyzer and commercially available kits. Patients’ levels of creatinine were measured by a blood test before and at the end of the study to evaluate the function of the kidney. 

Furthermore, the PBC-40 questionnaire validated for PBC and PSC (Wunsch et al., 2014 ▶), that evaluates QOL in terms of systemic, itching, fatigue, cognitive, social, and emotional aspects, was completed by each patient before and monthly during the study for three months. 

**Statistical analysis**

Statistical analysis was done by the software SPSS-16 (Chicago, SPSS Inc.; 2007, USA). Frequency Tables, mean± standard deviation, and medians with interquartile ranges (q25%–q75%) were used to describe the data. For creatinine results, the Wilcoxon test was used to compare changes before and after the study within the group. Given the nature of data for other measurements, the Friedman test was used to show the trend of changes within the group. The significance level in all tests was considered p<0.05.

## Results

**Total phenol content of** ***B. vulgaris***

The solid residue of the hydro alcoholic extract of *B. vulgaris* was 45% w/w. The content of total phenol in the extract was 105±12 mg of gallic acid equivalent per gram of the crude extract.

**Participant flow and demographic parameters**

**Figure 2:**
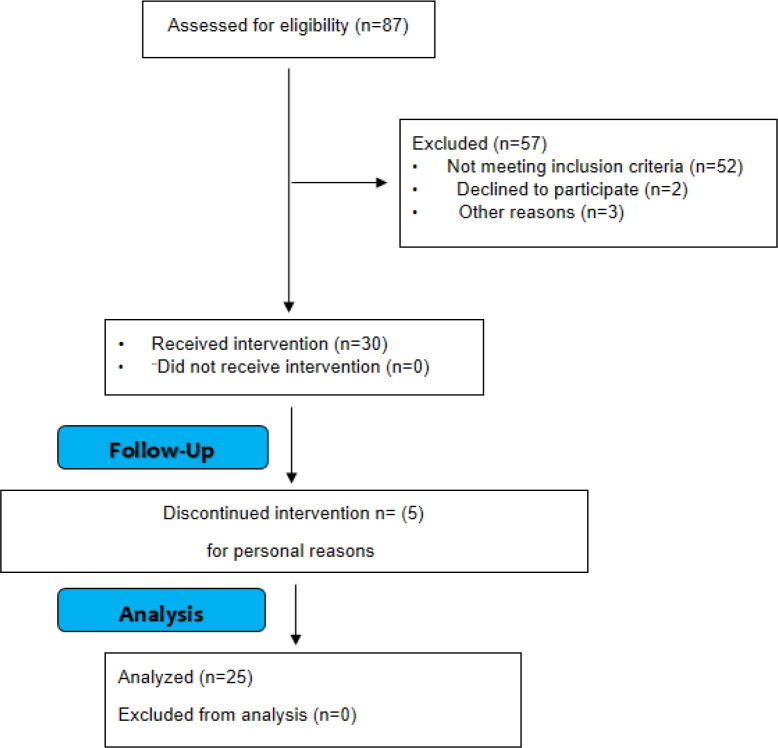
Consort flow for patients’ enrollment

From 87 patients referred to a gastroenterologist, 52 patients did not meet inclusion criteria and five patients refused to participate in the study due to personal reasons. Finally, 30 patients participated in the study (15 PSC patients and 15 PBC patients). During the study, two male patients with PSC and three females with PBC left the study for personal reasons (Figure 2). Finally, 25 patients completed the study. The demographic data of the patients who completed this study, are shown in Table 1.

**Table 1:** Demographic information of the participants

**Parameter**		**Total patients** **(PSC & PBC)**	**PSC** **patients**	**PBC** **patients**
Sex Number (percent)	Female	17 (%68)	5 (%38)	12 (%100)
	Male	8 (%32)	8 (%62)	0 (%0)
Age (year) (mean±SD^†^)		38.4±13.52	34.15±10.58	43±15.23

†SD: Standard deviation

**Effects of** ***B. vulgaris*** **oxymel on biochemical parameters**

**Liver associated enzyme levels in serum (ALP, AST, ALT, and GGT)**

**Figure 3:**
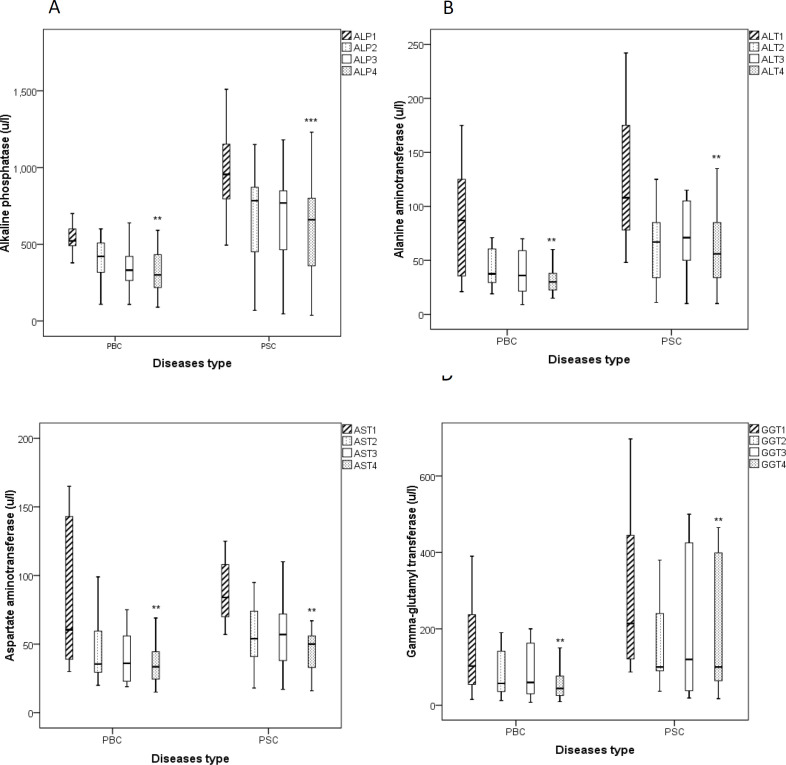
Boxplots show liver function-associated enzymes including alkaline phosphatase (A, ALP), alanine aminotransferase (B, ALT), aspartate aminotransferase (C, AST) and gamma glutamyl-transferase (D, GGT) in patients with PSC and PBC before and during the treatment with *B. vulgaris* oxymel. Data are presented as median± interquartile range. Friedman test was used as a statistical analysis test. 1: before study, 2: one month after initiation of the study, 3: two month after initiation of the study, 4: three month after initiation of the study, **p<0.01, and ***p<0.001; PBC: Primary Biliary Cholangitis, PSC: Primary Sclerosing Cholangitis

The level of ALP in both PBC and PSC groups treated with BO was significantly decreased after 3 month treatment in comparison to the beginning of the study (p<0.01 for PBC group and p<0.001 for PSC group, Figure 3A). Moreover, we found that the levels of ALT (Figure 3B), AST (Figure 3C), GGT (Figure 3D) were markedly attenuated following the BO treatment in both groups after three months compared to the enrolment day (p<0.05 for both cases). 

**Serum levels of total and direct bilirubin**

**Figure 4:**
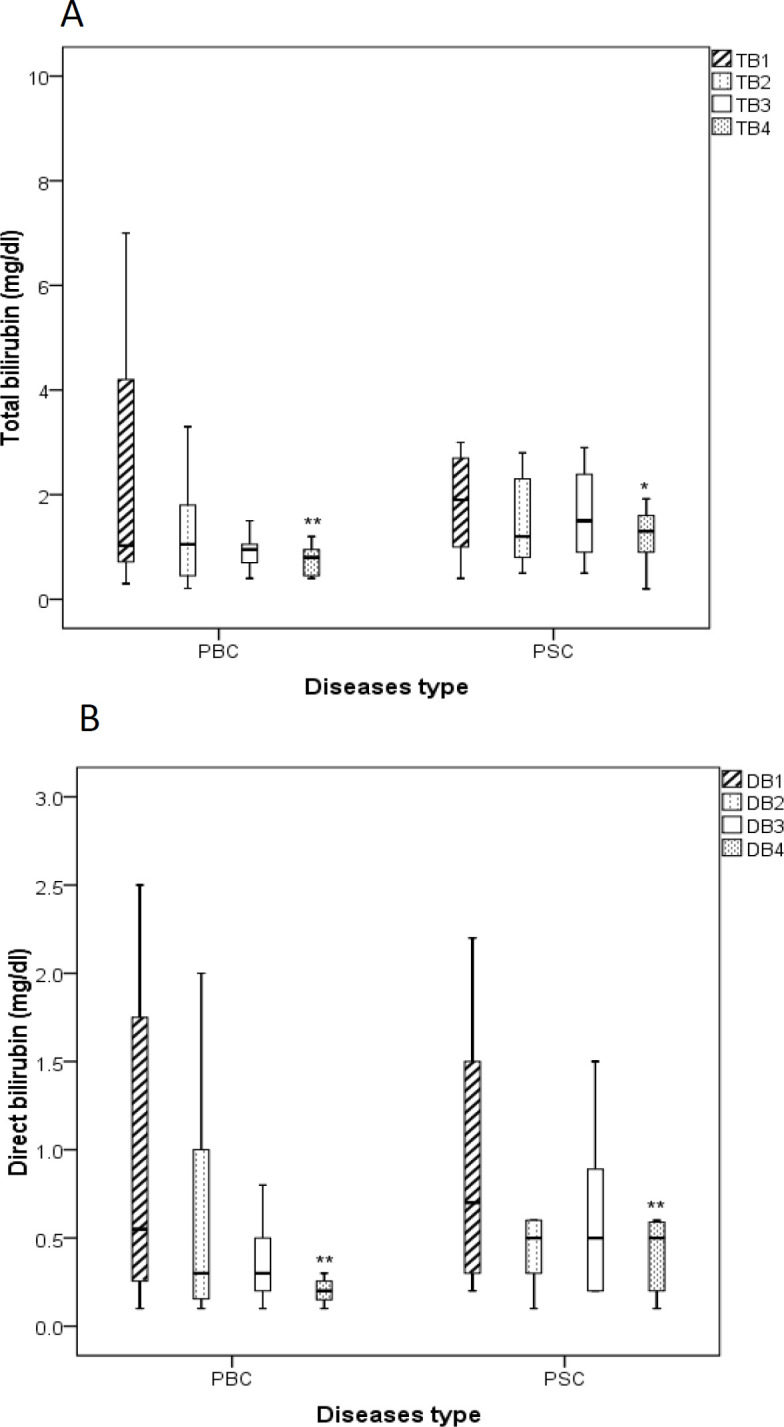
Boxplots show the effects of *B. vulgaris* oxymel medication on the levels of total bilirubin (A, TB) and direct bilirubin (B, DB) in patients with PSC and PBC before and during the treatment. Data are presented as median± interquartile range. Friedman test was used as a statistical analysis test. 1: before study, 2: one month after initiation of the study, 3: two month after initiation of the study, 4: three month after initiation of the study, *p<0.05, and **p<0.01; PBC: Primary Biliary Cholangitis, PSC: Primary Sclerosing Cholangitis

Our findings indicated that the levels of TB (Figure 4A) and DB (Figure 4B) were notably reduced in both PBC and PSC groups that received BO additional therapy after 3 months compared to the beginning of the study (p<0.05 for both cases). 

**Coagulation tests (PT and INR)**

**Figure 5:**
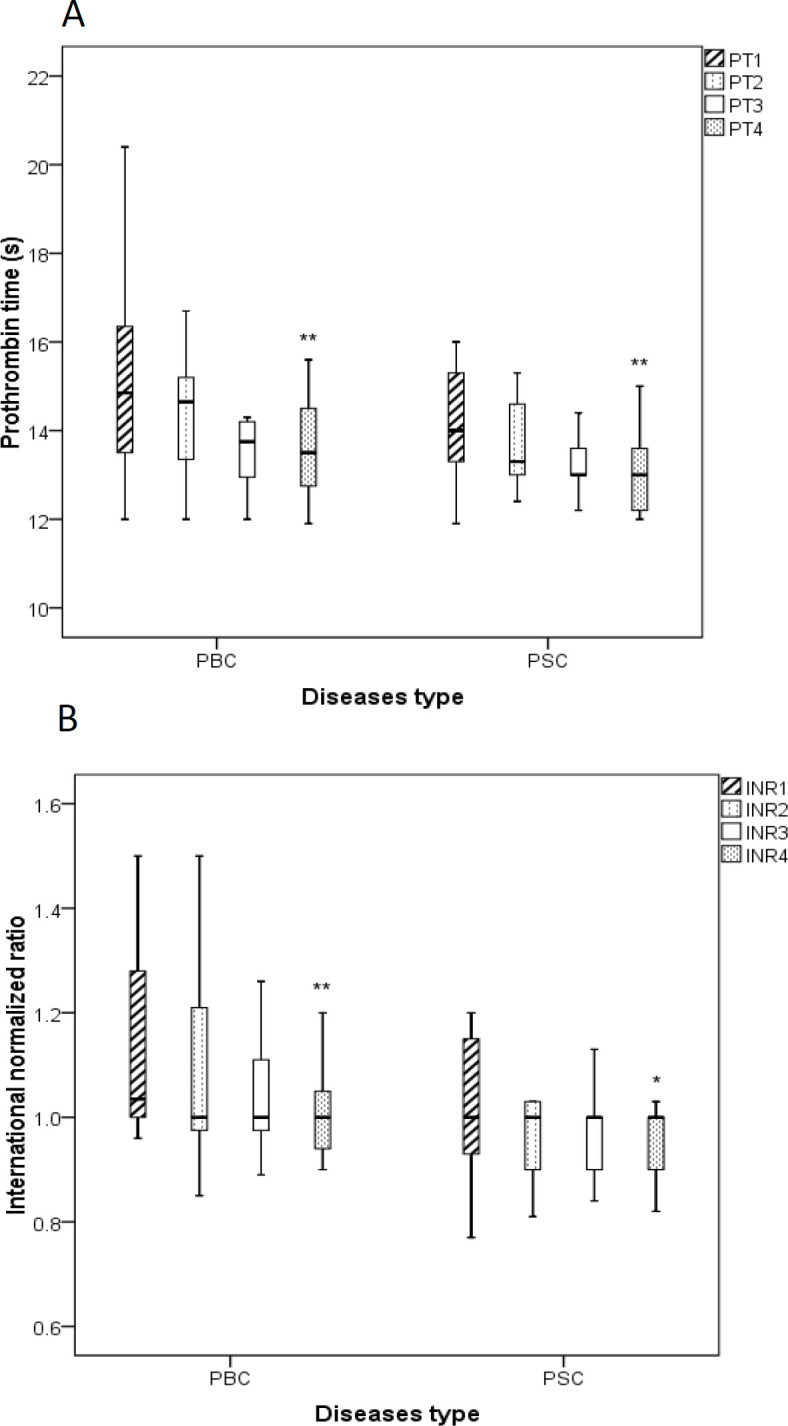
Boxplots show the effects of *B. vulgaris* oxymel medication on the levels of blood coagulation factors including prothrombin time (A, PT) and international normalized ratio (B, INR) in patients with PSC and PBC before and during the treatment. Data are presented as median± interquartile range. Friedman test was used as a statistical analysis test. 1: before study, 2: one month after initiation of the study, 3: two month after initiation of the study, 4: three month after initiation of the study, *p<0.05 and **p<0.01; PBC: Primary Biliary Cholangitis, PSC: Primary Sclerosing Cholangitis

The levels of PT (Figure 5A) and INR (Figure 5B), as known important coagulation markers, were meaningfully decreased in both PBC and PSC groups that received BO additional therapy after 3 months compared to the enrollment day (p<0.05 for both cases). 

**Serum levels of creatinine as a biomarker for renal function**

There was no significant change in creatinine level between before and at the end of the study in both PBC and PSC groups treated with BO (Table 2). 

**Table 2:** The level of creatinine (mg/dl) before and after the study

**Patients type**	**before study**	**after study**	**p-value**
	**Median (0.25 and 0.75 Quartiles)**	**Median (0.25 and 0.75 Quartiles)**	
PSC	0.9 (0.8, 1.05)	0.9 (0.8, 1.05)	0.52
PBC	1 (0.9,1.15)	1 (0.9, 1.08)	1

Wilcoxon test was used as the statistical analysis test. PSC: primary sclerosing cholangitis; PBC: primary biliary cholangitis

**Effects of** ***B. vulgaris*** **oxymel on** **QOL** **parameters**

**Figure 6:**
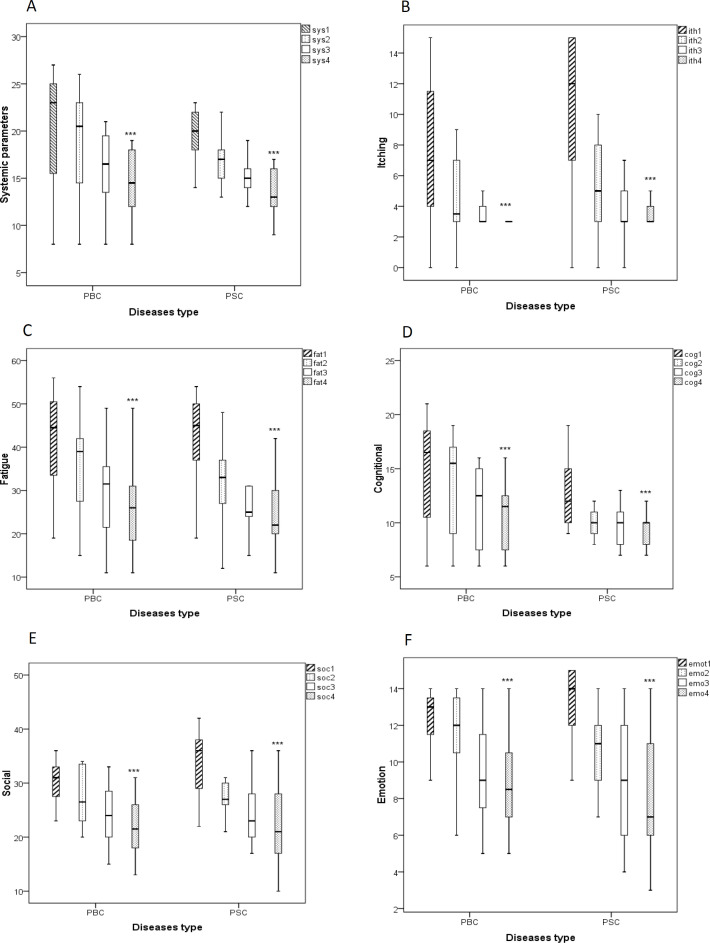
Boxplot of changes in the levels of quality of life patients with PBC and PSC based on the PBC-40 questionnaire. Data were presented as median± interquartile range. Friedman test was used as a statistical analysis test. sys: systemic parameters (A), itch: itching (B), fat: fatigue (C), cog: cognitional (D), soc: social (E), emo: emotion (F), PBC: primary biliary cholangitis, PSC: primary sclerosing cholangitis; 1: before study, 2: one month after initiation of the study, 3: two month after initiation of the study, 4: three month after initiation of the study, ***p<0.001

Evaluation of the effects of BO on QOL parameters including systemic parameters, fatigue, itching, social, cognition, and emotional symptoms showed that BO significantly improved the QOL following three-month treatment in comparison to the beginning of the study (p<0.001 for both cases, Figure 6).

## Discussion

To the best of our knowledge, this is the first clinical trial that investigates additional treatment of patients with chronic liver cholestasis with B. vulgaris oxymel. Briefly, our results indicated that the levels of AST (~50%), ALT (~40%), ALP (~35%), DB (~70%) and TB (~60%), GGT (~40%), PT (~10%), INR (~8%) were notably attenuated in patients with PBC and PSC receiving the BO for three months in comparison to the enrollment day. In this study, 6 out of 13 PSC patients (46% of the PSC patients in the study) and 7 out of 12 PBC patients (58% of the PBC patients in this study) who were given the BO showed about 40% reduction in the level of ALP compared to the baseline, or even reduction of ALP to less than 1.5 folds of the NUL.

Furthermore, we found that the medication of PBC and PSC patients with BO provides significant improvements in clinical and laboratory parameters.

In agreement with our findings, several animal and clinical reports indicated the therapeutic effects of *B. vulgaris* on hepatic disorders. In an animal study, it was shown that 200-600 mg/kg of *B. vulgaris* extract can reverse the liver damage induced by alloxan in diabetic rats. Authors reported that *B. vulgaris* extract attenuated the levels of glucose, serum glutamic-oxaloacetic transaminase, serum glutamic pyruvic transaminase, and ALP in comparison to the group that received only alloxan (Rahimi-Madiseh et al., 2017a ▶). In fact, by converting the effective applied dose to human dose (a man weighing 60 kg), the dose is between 1.2-3.6 g/d (average 2.4 g/d), which is similar to the dose we used for additional therapy in the present study. Therefore, it supports our study for the use of 2.2 g/d of *B. vulgaris* extract. Furthermore, in a recent clinical trial, the effect of *B. vulgaris* extract on transaminase activities in non-alcoholic fatty liver disease (NAFLD) was assessed in 80 patients. Authors found that the usage of 1.5 g of *B. vulgaris* aqueous extract daily can reduce the levels of ALT, AST, triglycerides, and cholesterol as well as liver lipid accumulation in comparison to the placebo-received group (Iloon Kashkooli et al., 2015 ▶). Indeed, the results of this study can support the findings of the present study, especially the dose of *B. vulgaris* extract seems about equal to the dose (2.2 g/d of *B. vulgaris* extract) we used in the present study.

Moreover, in this study, we evaluated the effects of BO additional therapy on clinical symptoms and QOL of patients with PBC and PSC using the PBC-40 questioner. These findings also demonstrated that systemic (~30%), fatigue (~35%), itching (~70%), emotional (~35%), social (~45%), and cognition (~35%) symptoms were markedly improved in both PBC and PSC groups after treatment with BO. 

In the present study, to evaluate the renal function, we assessed the levels of creatinine. We found that there were no significant changes during the consumption of BO when compared to the beginning of the study. In fact, this data can suggest that treatment with BO had no adverse effect on the kidney and all patients tolerated it very well. However, it seems necessary to evaluate further renal function parameters to certainly exclude its renal adverse effects. 

The BO is taken orally and the hydro alcoholic extract of *B. vulgaris* fruit has a bitter taste, but its aqueous extract has an agreeable taste and remarkable anti-oxidant and free radical scavenging properties (Motalleb et al., 2005 ▶); it is suggested that in future studies, BO should be prepared with aqueous extracts of the *B. vulgaris* fruit, due to its pleasant taste, which has a psychological effect in addition to its therapeutic effects. Therefore, in the present study and according to the *Qarabadin Kabir*’s instruction, we administrated the *B. vulgaris* fruit extract with grape vinegar and rose water. 

In the current study, grape vinegar was used to make oxymel. Several studies indicated that grape vinegar possesses a potent anti-oxidant and anti-inflammatory effects against different noxious stimuli (Dávalos et al., 2005 ▶; Samad et al., 2016 ▶). According to the satisfactory results of the present study, probably, the therapeutic effect of BO is not only dependent on the antioxidant and anti-inflammatory effect of *B. vulgaris* fruit but through increasing antioxidant and anti-inflammatory properties due to the combination of vinegar and *B. vulgaris* fruit. In fact, the positive interactive effects of the medicinal compositions of BO could strengthen the therapeutic effects of each other. 

As one of the limitations of the current study, no placebo group was considered in this study. In fact, we did not find it ethically that patients who were suffering from PBC and PSC as well as their adverse including itching, deprived from a possible treatment that may benefit them. However, the effects of BO on reducing itching in PSC and PBC were already and frequently reported in several folk medicines. As another limitation of our study, the enrolled patients who received BO additional therapy was deemed less, however, we performed the present study according to the statistically analyzed sample size. Therefore, considering the final results of the current study, doing similar random clinical trial studies with control groups and using various doses of BO for a longer duration can be suggested for further assessment in terms of efficacy and safety against PBC and PSC diseases. 

In summary, we showed that processed *B. vulgaris* fruit as an oxymel is effective in the treatment of patients with refractory PSC and PBC as well as improving the QOL symptoms and liver-related function laboratory tests. Moreover, we revealed that if this product is used at the therapeutic doses (about 2.2 g of *B. vulgaris* fruit extract), it has no adverse effect on the kidney. 

**Supplementary materials**

**Supplementary Table 1:** PBC-40 questionnaire (Wunsch et al., 2014)

*Can you say how often the following statements about digestion and diet applied to you IN THE LAST FOUR WEEKS?*												
1	I was able to eat what I liked	Never		Rarely		Sometimes		Most of the time		Always		
2	I ate or drank only a small amount, and still felt bloated	Never		Rarely		Sometimes		Most of the time		Always		
3	I felt unwell when I drank alcohol	Never		Rarely		Sometimes		Most of the time		Always		Did not apply /never drink alcohol
***And IN THE LAST FOUR WEEKS, how often did you experience any of the following?***												
4	I had discomfort in my right side	Never		Rarely		Sometimes		Most of the time		Always		
5	I had dry eyes	Never		Rarely		Sometimes		Most of the time		Always		
6	My mouth was very dry	Never		Rarely		Sometimes		Most of the time		Always		
7	I felt pain in the long bones of my arms and legs	Never		Rarely		Sometimes		Most of the time		Always		
***Some people with PBC experience itching. How often did you experience itching IN THE LAST FOUR WEEKS? If you did not itch, please circle ‘does not apply’***												
8	Itching disturbed my sleep	Never		Rarely		Sometimes		Most of the time		Always		Did not apply/ no itch
9	I scratched so much I made my skin raw	Never		Rarely		Sometimes		Most of the time		Always		Did not apply/no itch
10	I felt embarrassed because of the itching	Never		Rarely		Sometimes		Most of the time		Always		Did not apply/no itch
***Fatigue can also be a problem for many people with PBC. How often did the following statements apply to you IN THE LAST FOUR WEEKS?***												
11	I had to force myself to get out of bed		Never		Rarely		Sometimes		Most of the time		Always	
12	I had to have a sleep during the day		Never		Rarely		Sometimes		Most of the time		Always	
13	Fatigue interfered with my daily routine		Never		Rarely		Sometimes		Most of the time		Always	
14	I felt worn out		Never		Rarely		Sometimes		Most of the time		Always	
15	I felt so tired, I had to force myself to do the things I needed to do		Never		Rarely		Sometimes		Most of the time		Always	
16	I felt so tired, I had to go to bed early		Never		Rarely		Sometimes		Most of the time		Always	
17	Fatigue just suddenly hit me		Never		Rarely		Sometimes		Most of the time		Always	
18	PBC drained every ounce of energy out of me		Never		Rarely		Sometimes		Most of the time		Always	
***The next section is about the effort and planning that can be involved in living with PBC. Thinking about THE LAST FOUR WEEKS, how often did the following statements apply to you?***												
19	Some days it took me a long time to do things		Never		Rarely		Sometimes		Most of the time		Always	
20	If I was busy one day I needed at least another day to recover		Never		Rarely		Sometimes		Most of the time		Always	
21	I had to pace myself for day-to-day things		Never		Rarely		Sometimes		Most of the time		Always	
***The following statements are about the effects that PBC may have on things like memory and concentration. Thinking about THE LAST FOUR WEEKS, how often did the following statements apply to you?***												
22	Because of PBC I had to make a lot of effort to remember things		Never		Rarely		Sometimes		Most of the time		Always	
23	Because of PBC I had difficulty remembering things from one day to the next		Never		Rarely		Sometimes		Most of the time		Always	
24	My concentration span was short because of PBC		Never		Rarely		Sometimes		Most of the time		Always	
25	Because of PBC, I had difficulty keeping up with conversations		Never		Rarely		Sometimes		Most of the time		Always	
26	Because of PBC, I found it difficult to concentrate on anything		Never		Rarely		Sometimes		Most of the time		Always	
27	Because of PBC, I found it difficult to remember what I wanted to do		Never		Rarely		Sometimes		Most of the time		Always	
***Now some more general statements about how PBC may be affecting you as a person. How much do the following statements apply to you?***												
28	Because of PBC, I get more stressed about things than I used to		Not at all		A little		Somewhat		Quite a bit		Very much	
29	My sex life has been affected because of PBC		Not at all		A little		Somewhat		Quite a bit		Very much	
30	Having PBC gets me down		Not at all		A little		Somewhat		Quite a bit		Very much	
31	I feel I neglect my family because of having PBC		Not at all		A little		Somewhat		Quite a bit		Very much	
32	I feel guilty that I can’t do what I used to do because of having PBC		Not at all		A little		Somewhat		Quite a bit		Very much	
33	I worry about how my PBC will be in the future		Not at all		A little		Somewhat		Quite a bit		Very much	
***These statements relate to the possible effects of PBC on your social life. Thinking of your own situation, how much do you agree or disagree with them?***												
34	I sometimes feel so frustrated that I can not go out and enjoy myself		Strongly agree		Agree		Neither agree nor disagree		Disagree		Strongly disagree	
35	I tend to keep the fact that I have PBC to myself		Strongly agree		Agree		Neither agree nor disagree		Disagree		Strongly disagree	
36	I can’t plan holidays because of having PBC		Strongly agree		Agree		Neither agree nor disagree		Disagree		Strongly disagree	
37	My social life has almost stopped		Strongly agree		Agree		Neither agree nor disagree		Disagree		Strongly disagree	
***The next section is about the impact that PBC may be having on your life overall. How much do you agree or disagree with the following statements?***												
38	Everything in my life is affected by PBC		Strongly agree		Agree		Neither agree nor disagree		Disagree		Strongly disagree	
39	PBC has reduced the quality of my life		Strongly agree		Agree		Neither agree nor disagree		Disagree		Strongly disagree	
40	I can still lead a normal life, despite having PBC		Strongly agree		Agree		Neither agree nor disagree		Disagree		Strongly disagree	

## References

[B1] Abd AE-W, Ghareeb DA, Sarhan E, Abu- Serie MM, El Demellawy MA (2013). In vitro biological assessment of Berberis vulgaris and its active constituent, berberine: antioxidants, anti-acetylcholinesterase, anti-diabetic and anticancer effects. BMC complement and alternate med.

[B2] Angulo P, Jorgensen RA, Kowdley KV, Lindor KD (2008). Silymarin in the treatment of patients with primary sclerosing cholangitis: an open-label pilot study. Dig Dis Sci.

[B3] Angulo, Patel T, Jorgensen RA, Therneau TM, Lindor KD (2000). Silymarin in the treatment of patients with primary biliary cirrhosis with a suboptimal response to ursodeoxycholic acid. Hepatology.

[B4] Chapman R, Fevery J, Kalloo A, Nagorney DM, Boberg KM, Shneider B, Gores GJ (2010). Diagnosis and management of primary sclerosing cholangitis. Hepatology.

[B5] Corpechot C (2012). Primary biliary cirrhosis and bile acids. Clin Res Hepatol Gastroenterol.

[B6] Dávalos A, Bartolomé B, Gómez-Cordovés C (2005). Antioxidant properties of commercial grape juices and vinegars. Food chem.

[B7] Eaton JE, Nelson KM, Gossard AA, Carey EJ, Tabibian JH, Lindor KD, Larusso NF (2019). Efficacy and safety of curcumin in primary sclerosing cholangitis: an open label pilot study. Scand J Gastroenterol.

[B8] Ghorbani A, Mohebbati R, Jafarian AH, Vahedi MM, Hosseini SM, Soukhtanloo M, Sadeghnia HR (2016). Toxicity evaluation of hydroalcoholic extract of Ferula gummosa root. Regul Toxicol Pharmacol.

[B9] Hanachi P, Kua S, Asmah R, Motalleb G, Fauziah O (2006). Cytotoxic effect of Berberis vulgaris fruit extract on the proliferation of human liver cancer cell line (HepG2) and its antioxidant properties. Int J cancer res.

[B10] Hosseini A, Mollazadeh H, Amiri MS, Sadeghnia HR, Ghorbani A (2017). Effects of a standardized extract of Rheum turkestanicum Janischew root on diabetic changes in the kidney, liver and heart of streptozotocin-induced diabetic rats. Biomed Pharmacother.

[B11] Kashkooli R, Najafi SS, Sharif F, Hamedi A, Hoseini As, Najafi Kalyani M, Birjandi M (2015). The effect of berberis vulgaris extract on transaminase activities in non-alcoholic Fatty liver disease. Hepat Mon.

[B12] Imanshahidi M, Hosseinzadeh H (2008). Pharmacological and therapeutic effects of Berberis vulgaris and its active constituent, berberine. Phytother Res.

[B13] Khorasani SA (1859). Gharabadin kabir, Tehran, Institute for the Study of Medical History and Islamic Medicine and compelmentary medicine.

[B14] Kowdley KV, Luketic V, Chapman R, Hirschfield GM, Poupon R, Schramm C, Vincent C, Rust C, Pares A, Mason A (2018). A randomized trial of obeticholic acid monotherapy in patients with primary biliary cholangitis. Hepatology.

[B15] Lazaridis KN, Gores GJ, Lindor KD (2001). Ursodeoxycholic acid ‘mechanisms of action and clinical use in hepatobiliary disorders. J Hepatol.

[B16] Lindor KD, Gershwin ME, Poupon R, Kaplan M, Bergasa NV, Heathcote EJ (2009). Primary biliary cirrhosis. Hepatology.

[B17] Milkiewicz P, Wunsch E, Elias E (2012). Liver transplantation in chronic cholestatic conditions. Front Biosci.

[B18] Mirazi N, Tohidi F, Hosseini A (2015). Effect of berberis vulgaris hydroethanolic extract on serum bilirubin level and liver enzymes in male cholstatic rats. Knowledge and Health.

[B19] Motalleb G, Hanachi P, Kua S, Fauziah O, Asmah R (2005). Evaluation of phenolic content and total antioxidant activity in Berberis vulgaris fruit extract. J Biol Sci.

[B20] Pisonero-Vaquero S, González-Gallego J, Sánchez-Campos S, Victoria Garcia-Mediavilla M (2015). Flavonoids and related compounds in non-alcoholic fatty liver disease therapy. Curr Med Chem.

[B21] Rahimi-Madiseh M, Karimian P, Kafeshani M, Rafieian-Kopaei M (2017a). The effects of ethanol extract of Berberis vulgaris fruit on histopathological changes and biochemical markers of the liver damage in diabetic rats. Iran J Basic Med Sci.

[B22] Rahimi-Madiseh M, Lorigoini Z, Zamani-Gharaghoshi H, Rafiean-Kopaei M (2017b). Berberis vulgaris: specifications and traditional uses. Iran J Basic Med Sci.

[B23] Rodriguez -Ramiro I, Vauzour D, Minihane A (2016). Polyphenols and non-alcoholic fatty liver disease: impact and mechanisms. Proc Nutr Soc.

[B24] Samad A, Azlan A, Ismail A (2016). Therapeutic effects of vinegar: a review. Curr Opin Food Sci.

[B25] Wunsch E, Trottier J, Milkiewicz M, Raszeja-Wyszomirska, Hirschfiled GM, Barbier O, Milkiewicz P (2014). Prospective evaluation of ursodeoxycholic acid withdrawal in patients with primary sclerosing cholangitis. Hepatology.

[B26] Zarei A, Changiz-Ashtiyani S, Taheri S, Ramezani M (2015). A quick overview on some aspects of endocrinological and therapeutic effects of Berberis vulgaris L. Avicenna J Phytomed.

[B27] Zargaran A, Zarshenas M, Mehdizadeh A, Mohagheghzadeh A (2012). Oxymel in medieval Persia. Pharm Hist (Lond).

